# CD8^+^ T cells undergo activation and programmed death-1 repression in the liver of aged *Ae2_a,b_^−/−^* mice favoring autoimmune cholangitis

**DOI:** 10.18632/oncotarget.5665

**Published:** 2015-09-15

**Authors:** Axel R. Concepcion, January T. Salas, Elena Sáez, Sarai Sarvide, Alex Ferrer, Ainhoa Portu, Iker Uriarte, Sandra Hervás-Stubbs, Ronald P.J. Oude Elferink, Jesús Prieto, Juan F. Medina

**Affiliations:** ^1^ Center for Applied Medical Research (CIMA), School of Medicine and Clinic University of Navarra, and Ciberehd, Pamplona, Spain; ^2^ Department of Pathology and Laboratory Medicine, University of Pennsylvania Perelman School of Medicine, Philadelphia, PA, USA; ^3^ Department of Immunology, Mayo Clinic College of Medicine, Rochester, MN, USA; ^4^ Tytgat Institute for Liver and Intestinal Research, Academic Medical Center, Amsterdam, The Netherlands

**Keywords:** Na^+^-independent Cl^−^/HCO3^−^ anion exchanger AE2, mouse model of autoimmune cholangitis, intracellular pH homeostasis, age-related changes, self-tolerance breakdown, Immunology and Microbiology Section, Immune response, Immunity

## Abstract

Primary biliary cirrhosis (PBC) is a chronic cholestatic disease of unknown etiopathogenesis showing progressive autoimmune-mediated cholangitis. In PBC patients, the liver and lymphocytes exhibit diminished expression of AE2/SLC4A2, a Cl^−^/HCO_3_^−^ anion exchanger involved in biliary bicarbonate secretion and intracellular pH regulation. Decreased AE2 expression may be pathogenic as *Ae2_a,b_^−/−^* mice reproduce hepatobiliary and immunological features resembling PBC. To understand the role of AE2 deficiency for autoimmunity predisposition we focused on the phenotypic changes of T cells that occur over the life-span of *Ae2_a,b_^−/−^* mice. At early ages (1-9 months), knockout mice had reduced numbers of intrahepatic T cells, which exhibited increased activation, programmed-cell-death (PD)-1 expression, and apoptosis. Moreover, young knockouts had upregulated PD-1 ligand (PD-L1) on bile-duct cells, and administration of neutralizing anti-PD-L1 antibodies prevented their intrahepatic T-cell deletion. Older (≥10 months) knockouts, however, showed intrahepatic accumulation of cytotoxic CD8^+^ T cells with downregulated PD-1 and diminished apoptosis. *In-vitro* DNA demethylation with 5-aza-2′-deoxycytidine partially reverted PD-1 downregulation of intrahepatic CD8^+^ T cells from aged knockouts. Conclusion: Early in life, AE2 deficiency results in intrahepatic T-cell activation and PD-1/PD-L1 mediated deletion. With aging, intrahepatic CD8^+^ T cells epigenetically suppress PD-1, and their consequential expansion and further activation favor autoimmune cholangitis.

## INTRODUCTION

Primary biliary cirrhosis (PBC) is a chronic cholestatic disease resulting from immune-mediated destruction of intrahepatic bile ducts [1-3]. The disease is characterized by development of serum antimitochondrial antibodies (AMA) and the presence of portal inflammation with autoreactive T lymphocytes, particularly CD8^+^ T cells, surrounding the interlobular bile ducts [4]. The peptide sequences of the major self-antigens recognized by AMA and autoreactive T cells are contained in the inner lipoyl domain of the E2 subunit of the mitochondrial pyruvate dehydrogenase complex (PDC-E2) [5, 6]. On the other hand, lymphocytes in the liver and peripheral blood of PBC patients were reported to have lower proportion of regulatory T cells (Tregs) than expected when compared with suitable controls [7].

Despite prominent autoimmune phenomena in PBC, classical immunosuppressants are therapeutically ineffective, while ursodeoxycolic acid (a bile acid that induces bicarbonate-rich hydrocholeresis) provides significant benefits in most PBC patients when initiated early during the evolution of the disease [8]. Our previous and current studies support the notion that alterations in the mechanisms of HCO_3_^−^ transport in cholangiocytes and immunocytes may play a pathogenic role in PBC (see recent reviews in ref. [9-11]). Biliary bicarbonate secretion is mainly exerted by AE2 (also termed SLC4A2), a Cl^−^/HCO_3_^−^ anion exchanger known to be also involved in intracellular pH (pH_i_) regulation in many cell types [11, 12]. AE2 expression is diminished in both cholangiocytes and peripheral blood lymphocytes (PBLs) from PBC patients [9, 11, 13]. Resulting impaired biliary HCO_3_^−^ transport [14] may hinder the formation of the “bicarbonate umbrella” that protects the apical membrane of cholangiocytes against bile-salt induced injury [15], and lead to damaged cholangiocytes that expose self-antigens to immune attack. In lymphocytes, AE2 deficiency may cause elevation of pH_i_ and enhanced reactivity [16]. Our recent data in *Ae2_a,b_^−/−^* mice indicate that CD4^+^ T cells can express AE1 in addition to AE2, whereas CD8^+^ T cells rely on AE2 as the only acidifying mechanism to maintain pH_i_ within physiological values [16]. Noticeably, AE2_a,b_-deficient CD8^+^ T cells exhibit excessive intracellular alkalinization and enhanced expansion upon T-cell stimulation [16].

PBC typically occurs in middle-aged women and more rarely in young individuals. Similarly *Ae2_a,b_^−/−^* mice develop immune-mediated cholangitis in adult age [17]. The reason why autoimmunity develops at later stages of life remains unknown. In the present study, we found that in young *Ae2_a,b_^−/−^* mice CD8^+^ T cells become activated in the liver but are deleted by apoptosis mediated by PD-1/PD-L1 interaction. In older *Ae2_a,b_^−/−^* mice, however, epigenetic silencing of PD-1 in activated intrahepatic CD8^+^ T cells prevents their apoptotic deletion with resulting cell expansion and autoimmune bile duct damage. Our findings illuminate the role of AE2 for immune homeostasis and reveal that deficiency of AE2 in liver-infiltrating CD8^+^ T cells may lead to age-related epigenetic changes affecting immunosuppressive mechanisms that contribute to autoimmunity.

## RESULTS

### Progressive changes in intrahepatic and peripheral T lymphocytes of *Ae2_a,b_^−/−^* mice

**Figure 1:**
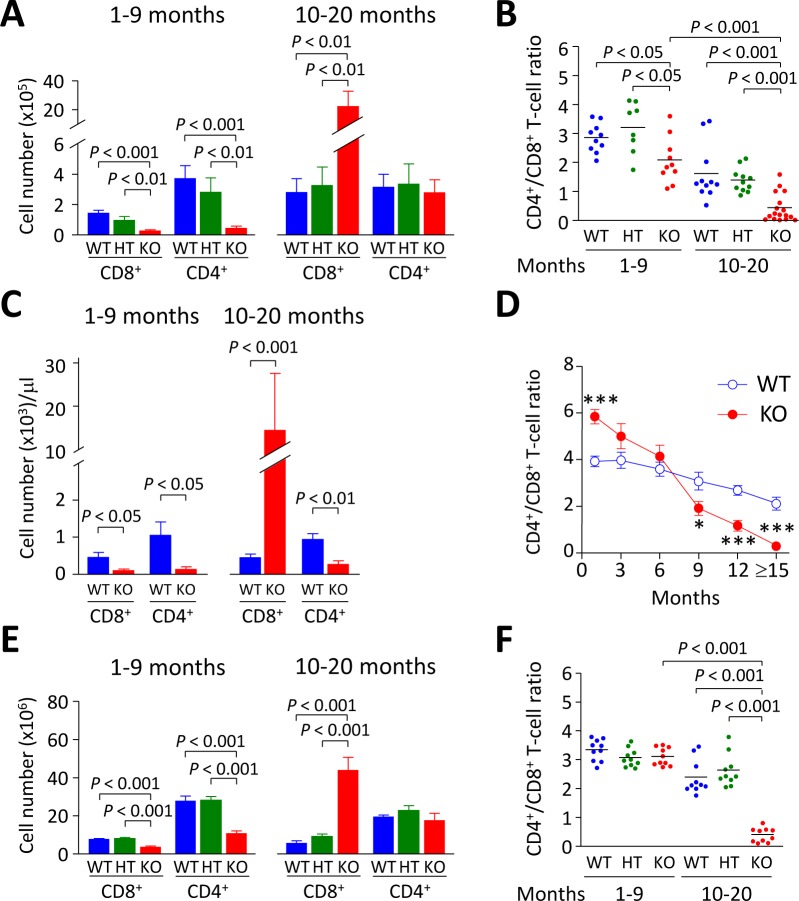
CD8^+^ T cells accumulate steadily with aging in *Ae2_a,b_^−/−^* mice **A.** Cell number of liver-infiltrating CD8^+^ and CD4^+^ T lymphocytes of young (1-9 month old) and aged (10-20 month old) WT, *Ae2_a,b_^+/−^* (HT), and *Ae2_a,b_^−/−^* (KO) mice. **B.** Intrahepatic CD4^+^/CD8^+^ T-cell ratio in mice as in (A). **C.** Number of CD8^+^ and CD4^+^ T cells in peripheral blood of both young and aged *Ae2_a,b_^−/−^* and WT mice. **D.** Follow-up of the CD4^+^/CD8^+^ T-cell ratio in blood of *Ae2_a,b_^−/−^* and WT mice at different ages. **E.** Number of CD8^+^ and CD4^+^ T cells and **F.** CD4^+^/CD8^+^ T-cell ratio in the spleen of mice as in A. Data are shown as mean ± SEM of *n* = 8 mice in A, 5 in C and 10 in E, per genotype and group. In B and F, dots indicate individual values and bars are mean values. *P < 0.05, and ***P < 0.001.

**Figure 2:**
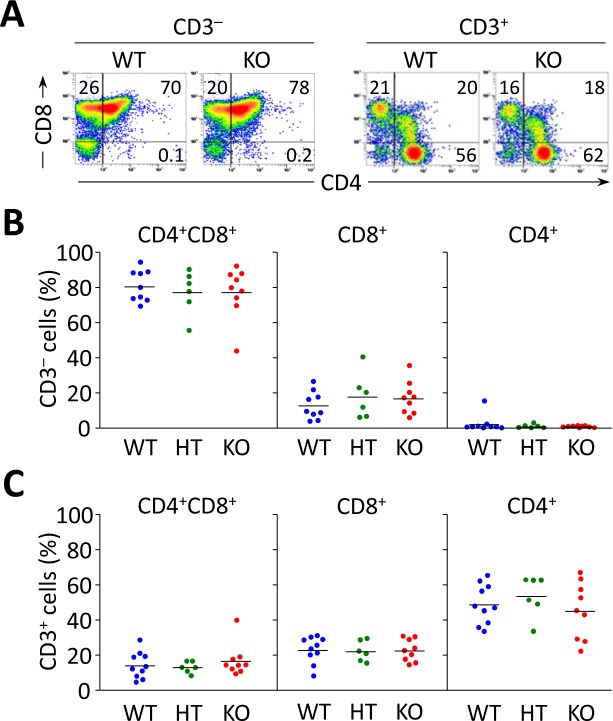
Flow cytometry analyses of thymocyte subsets in *Ae2_a,b_^−/−^* mice up to 10 months show no differences compared to littermate controls **A.** Representative density plots showing the CD3^−^ and CD3^+^ thymocyte subsets of WT, *Ae2_a,b_^+/−^* (HT), and *Ae2_a,b_^−/−^* (KO) mice. **B.** and **C.** Percentage of double-positive CD4^+^CD8^+^ and single positive CD4^+^ and CD8^+^ into CD3^−^ (in B) and CD3^+^ populations (in C). Each dot represents the value for an individual mouse, and horizontal bars represent mean values.

Analysis of liver-infiltrating CD8^+^ and CD4^+^ T lymphocytes showed decreased cell numbers in young *Ae2_a,b_^−/−^* mice (1-9 months of age) compared to WT and HT littermates (Figure 1A). At older age (10-20 months), however, *Ae2_a,b_^−/−^* mice had markedly increased intrahepatic CD8^+^ (but not CD4^+^) T cells (Figure 1A), and inverted CD4^+^/CD8^+^ T-cell ratio (Figure 1B). Similarly to the liver, young *Ae2_a,b_^−/−^* mice manifested reduced T-cell numbers in blood and spleen, while aged knockouts showed robust expansion of circulating and splenic CD8^+^ (but not CD4^+^) T cells (Figure 1C-1F). Noticeably, the circulating CD4^+^/CD8^+^ T-cell ratio shifted over time from an initial increase in 1-month old knockouts to reduction and inversion in ≥15-month old *Ae2_a,b_^−/−^* mice *versus* WT littermates (Figure 1D). These changes are seemingly unrelated to defects in T-cell development, as analysis of the thymus in *Ae2_a,b_^−/−^* mice (up to 10-month old) showed no abnormalities in CD8^+^, CD4^+^, and double positive (CD4^+^CD8^+^) thymocytes (Figure 2).

**Figure 3:**
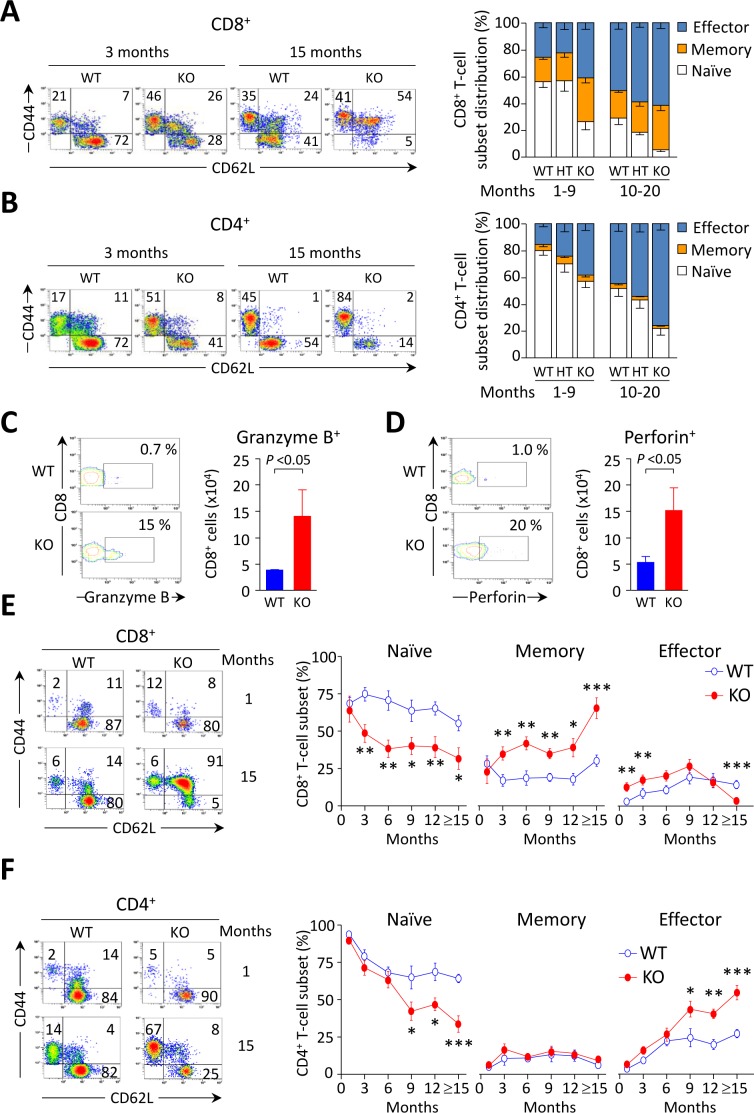
CD8^+^ T cells are activated at early ages in the liver of *Ae2_a,b_^−/−^* mice **A.** and **B.** Representative density plots (left) and distribution of T-cell subsets (right), *i.e.* naïve (CD44^lo^CD62L^hi^), memory (CD44^hi^CD62L^hi^) and effector (CD44^hi^CD62L^lo^) T cells, illustrating the activation status of intrahepatic CD8^+^ (in A) and CD4^+^ (in B) T cells in young and old mice of the three genotypes. **C.** and **D.** Representative contour plot (left) and total number (right) of intrahepatic CD8^+^ T cells stained for intracellular granzyme B (in C) and perforin (in D) in aged mice. **E.** and **F.** Representative density plots of the activation status of CD8^+^ (in E) and CD4^+^ T cells (in F) in peripheral blood of WT and *Ae2_a,b_^−/−^* mice at the indicated ages (left), and follow-up of the respective subsets (right) in blood of WT and *Ae2_a,b_^−/−^* mice along the time. Results are shown as mean ± SEM of *n =* 7-9 mice per genotype and age (but *n =* 5 in C and D). *P < 0.05, **P < 0.01, and ***P < 0.001.

Young *Ae2_a,b_^−/−^* mice exhibited early activation of T cells in the liver, with increased proportions of memory and effector subsets (Figure 3A, 3B, and Table 1). In aged knockouts, intrahepatic T-cell activation was further accelerated, particularly in the CD8^+^ population which almost lacked a naïve subset (Figure 3A, 3B) and upregulated the expression of the cytotoxic molecules granzyme B and perforin (Figure 3C, 3D). Also circulating CD8^+^ T cells manifested early activation in *Ae2_a,b_^−/−^* mice, with obvious differences *versus* WT littermates at 1 and 3 months of age (Figure 3E), whereas the enhanced activation of circulating CD4^+^ T cells of the knockouts proceeded more slowly (Figure 3F).

**Table 1:** *P* values for differences between liver-infiltrating T-lymphocyte subsets in *Ae2_a,b_^−/−^* and WT mice at different ages

	Intrahepatic CD8^+^ T cells			Intrahepatic CD4^+^ T cells		
	Naïve	Memory	Effector	Naïve	Memory	Effector
**1-9 months**						
KO *vs* WT	*P* = 0.0017	*P* = 0.0073	*P* = 0.0262	*P* = 0.0049	N.S.	*P* = 0.0024
KO *vs* HT	*P* = 0.0122	*P* = 0.0409	*P* = 0.0279	N.S.	N.S.	N.S.
**1-9 months**						
KO *vs* WT	*P =* 0.0003	*P* = 0.0034	*P* = 0.0236	*P* = 0.0026	N.S.	*P* = 0.0015
KO *vs* HT	*P* = 0.0006	N.S.	N.S.	*P* = 0.0093	N.S.	*P* = 0.0093

N.S. Not significant

### Progressive activation and expansion of CD8^+^ T cells runs parallel with increasing pH_i_ of PBLs in *Ae2_a,b_^−/−^* mice

**Figure 4:**
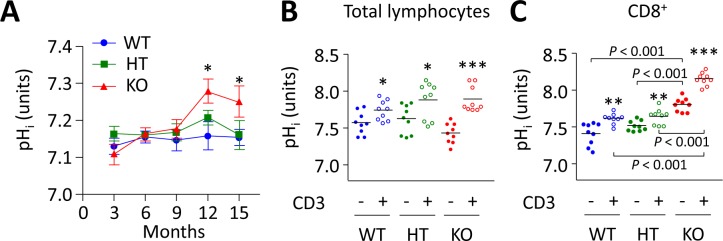
Intracellular alkalinization of PBLs in *Ae2_a,b_^−/−^* mice progresses over time and runs parallel with both expansion and activation of CD8^+^ T cells **A.** Follow up of pH_i_ in total lymphocytes isolated from peripheral blood of WT, HT, and *Ae2_a,b_^−/−^* mice at different ages. Results are shown as mean ± SEM of at least 10 mice per genotype and age. *P < 0.05 *versus* WT littermates. **B.** and **C.** pH_i_ values of total lymphocytes (in B) and purified CD8^+^ T cells (in C) isolated from the spleen of young WT, HT, and *Ae2_a,b_^−/−^* mice and cultured in complete RPMI medium for 1 day in the presence or absence of anti-CD3 (1 *μ*g/mL). Each dot represents the value for an individual mouse, and horizontal bars represent mean values. *P < 0.05, **P < 0.01, and ***P < 0.001 *versus* unstimulated conditions.

**Figure 5:**
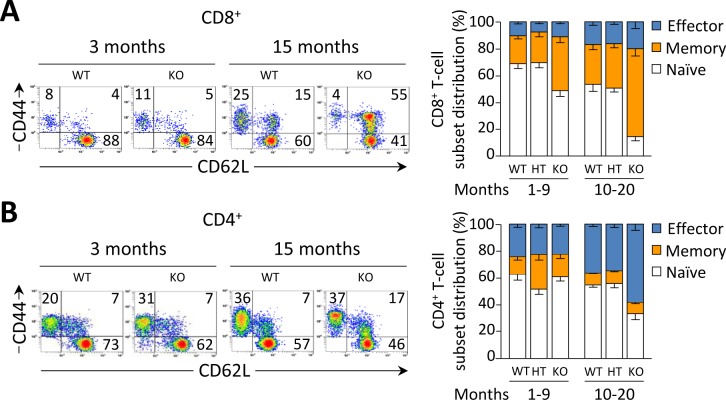
Activation status of T cells in the spleen of WT, HT, and *Ae2_a,b_^−/−^* mice analyzed by flow cytometry **A.** and **B.** Representative density plots (left) and T-cell subset distributions (right), *i.e.* naïve (CD44^lo^CD62L^hi^), memory (CD44^hi^CD62L^hi^) and effector (CD44^hi^CD62L^lo^) T cells, illustrating the activation status of CD8^+^ (in A) and CD4^+^ T splenocytes (in B) in both young and old mice. Data are shown as mean ± SEM of *n* = 8 mice per genotype and group.

AE2-mediated HCO_3_^−^ extrusion is known to be important in several cell types for the regulation of pH_i_ following cytosolic alkalinization [11, 12]. Concerning CD8^+^ T cells we recently reported that they are strictly dependent on AE2 to maintain pH_i_ homeostasis after activation, and that CD8^+^ splenocytes from young *Ae2_a,b_^−/−^*mice exhibit intracellular alkalinization [16]. Now we found in peripheral blood that the basal pH_i_ of PBLs is increased only in aged *Ae2_a,b_^−/−^* mice but not in young knockouts (Figure 4A), the latter being seemingly due to the initial low proportion of CD8^+^ T cells in PBLs. Indeed, pH_i_ measurements in the spleen of young knockouts revealed that, at baseline, only purified CD8^+^ T cells but not total splenocytes have intracellular alkalinization, while following T-cell activation both CD8^+^ T cells and total splenocytes increased the pH_i_ (Figure 4B, 4C). In contrast to young knockouts, aged *Ae2_a,b_^−/−^* mice (10-20 month old) showed baseline intracellular alkalinization of total splenocytes (in agreement with our previous observation in 15-month old *Ae2_a,b_^−/−^* mice) [17], which reflects the aforementioned expansion of CD8^+^ T cells that colonizes and enlarges the spleen in aged knockouts (Supplementary Figure S1A, S1B). This late global intracellular alkalinization also reflects the increased activation (and consequent increased pH_i_) of both CD8^+^ and CD4^+^ T cells in the spleen of aged *Ae2_a,b_^−/−^* mice, that contrasts with the minor increase in intrasplenic memory CD8^+^ T-cell subset in young knockouts (Figure 5 and Supplementary Figure S1C). Altogether, our data support the notion that in *Ae2_a,b_^−/−^* mice T cells are activated early in the liver and subsequently memory and effector T cells also appear in peripheral blood and spleen in addition to the liver.

### PD-1/PD-L1 interaction mediates the deletion of activated intrahepatic CD8^+^ T cells in young *Ae2_a,b_^−/−^* mice

**Figure 6:**
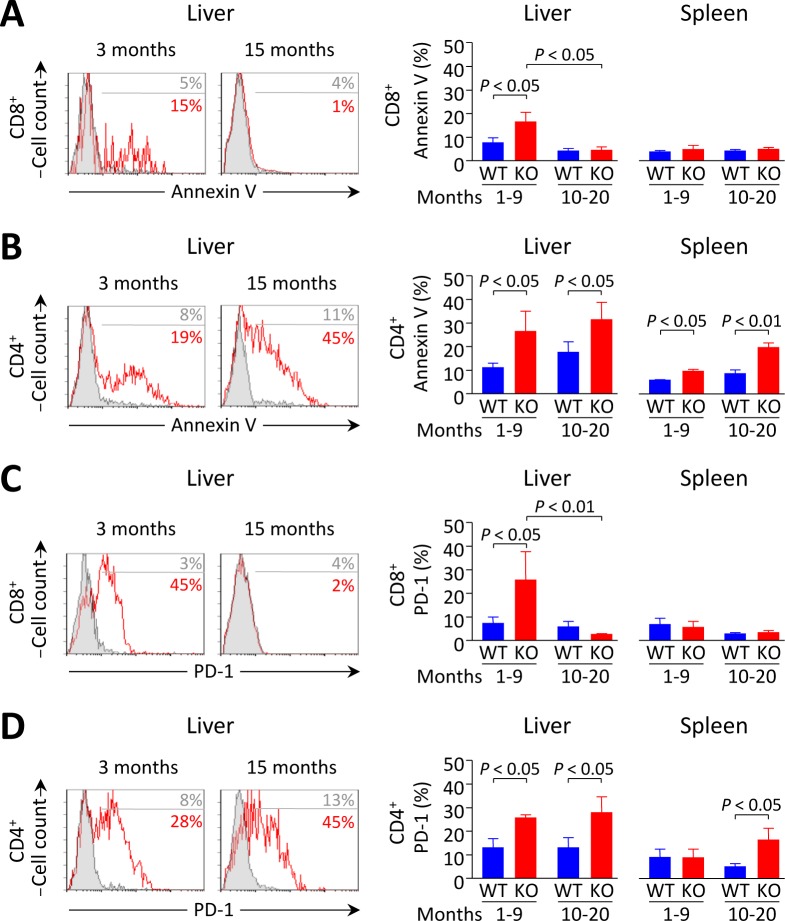
Increased apoptosis and PD-1 expression in liver-infiltrating CD8^+^ T cells from *Ae2_a,b_^−/−^* mice recede with aging **A.** and **B.** Apoptotic rate measured by annexin-V staining in CD8^+^ (in A) and CD4^+^ T cells (in B) in young and aged mice: representative flow-cytometry histograms (left) and percentages (right) of annexin-V^+^ intrahepatic T cells (and also intrasplenic T cells for the percentages). **C.** and **D.** Flow-cytometry analysis of PD-1 on CD8^+^ (in C) and CD4^+^ T cells (in D) in the same samples as in A and B, respectively. Results are shown as mean ± SEM of *n =* 4 young mice per genotype and *n =* 5 aged mice per genotype.

In the liver, terminal differentiation/activation of lymphocytes was reported to result in apoptotic cell deletion through PD-1/PD-L1 interaction [18]. We therefore hypothesized that the reduction in the number of intrahepatic T cells observed in young knockouts might be due to accelerated cell death within the liver. Indeed we found that the apoptotic rate of liver-infiltrating T cells (as manifested by annexin-V expression) was increased in young *Ae2_a,b_^−/−^* mice (Figure 6A, 6B), while a particularly diminished apoptotic rate occurred in intrahepatic CD8^+^ T cells of aged *Ae2_a,b_^−/−^* mice (Figure 6A). Notably, the percentage of PD-1^+^ intrahepatic CD8^+^ T cells was greatly increased in young knockouts while PD-1 expression decreased sharply in old *Ae2_a,b_^−/−^* mice (Figure 6C). In contrast to these age-related changes in annexin-V and PD-1 expression on intrahepatic CD8^+^ T cells, elevated frequencies of annexin-V^+^ and PD-1^+^ cells were found in intrahepatic CD4^+^ T cells of both young and aged *Ae2_a,b_^−/−^*mice (Figure 6B, 6D). On the other hand, CD8^+^ T cells never increased the expression of these molecules in both the spleen and peripheral blood regardless the genotype and the age of the animals (Figure 6 and Supplementary Figure S2).

**Figure 7:**
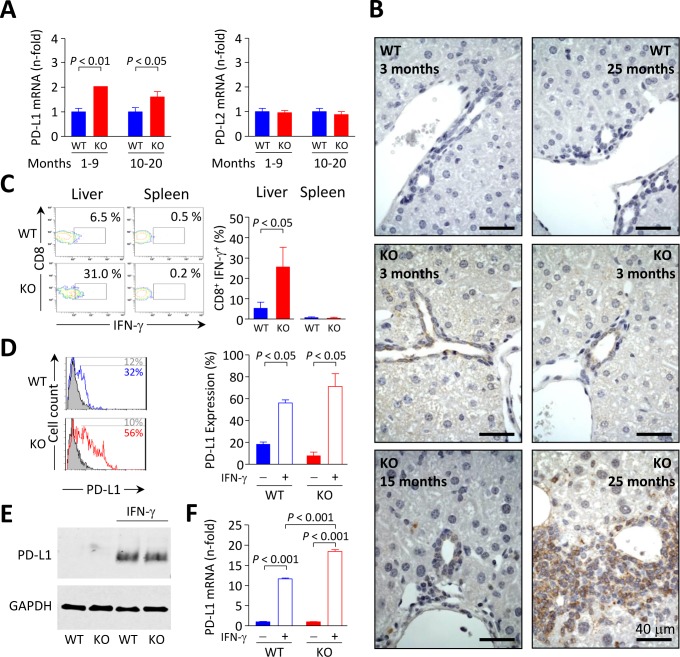
PD-L1 expression in mouse biliary epithelial cells is influenced by IFN-γ **A.** Relative PD-L1 (left) and PD-L2 (right) mRNA levels measured by qPCR in liver samples from *Ae2_a,b_^−/−^* and WT mice. Results are shown as mean ± SEM of *n =* 5 mice per genotype and group. **B.** Immunohistochemical staining of PD-L1 on liver sections of *Ae2_a,b_^−/−^* and WT mice at the indicated ages. **C.** Representative contour plot (left) and percentages (right) of IFN-γ^+^ cells gated on intrahepatic and intrasplenic CD8^+^ T cells from young *Ae2_a,b_^−/−^* mice and WT littermates. Results are shown as mean ± SEM of *n =* 4 young mice per genotype. **D.** Representative flow-cytometry histograms (left) and percentages (right) of PD-L1 expression on isolated cholangiocytes from *Ae2_a,b_^−/−^* and WT mice cultured for 2 days with (open histograms) or without (full histograms) 100 ng/mL IFN-γ. **E.** Representative Western blot showing the expression of PD-L1 in cultured *Ae2_a,b_^−/−^* and WT mouse cholangiocytes treated as in D. **F.** Levels of PD-L1 mRNA (measured by qPCR, with GAPDH as the normalizing control) in cultured *Ae2_a,b_^−/−^* and WT mouse cholangiocytes treated as in D. Results are pooled from 4 independent experiments.

PD-L1 and PD-L2 are the natural ligands for negative lymphocyte regulation through PD-1 signaling. Using qPCR we found that PD-L1 (but not PD-L2) mRNA levels are enhanced in the liver of *Ae2_a,b_^−/−^* mice (Figure 7A). Immunohistochemical staining of PD-L1 in the liver of young animals showed absence of this molecule in WT but detectable levels in bile ducts of *Ae2_a,b_^−/−^* mice (Figure 7B). Noticeably, the liver (but not the spleen) of these young knockouts had an increased frequency of CD8^+^ T cells expressing interferon (IFN)-γ (Figure 7C), a cytokine which has been previously reported to trigger PD-L1 expression in cultured human cholangiocytes [19]. In full agreement with these findings in human bile-duct cells [19] we observed that IFN-γ upregulates PD-L1 (both mRNA and protein) in cultured mouse cholangiocytes regardless their genotype (Figure 7D-7F). PD-L1 immunostaining in the liver of aged *Ae2_a,b_^−/−^* mice with cholangitis, revealed PD-L1 expression not only in bile-ducts but also in surrounding inflammatory cells (Figure 7B).

**Figure 8:**
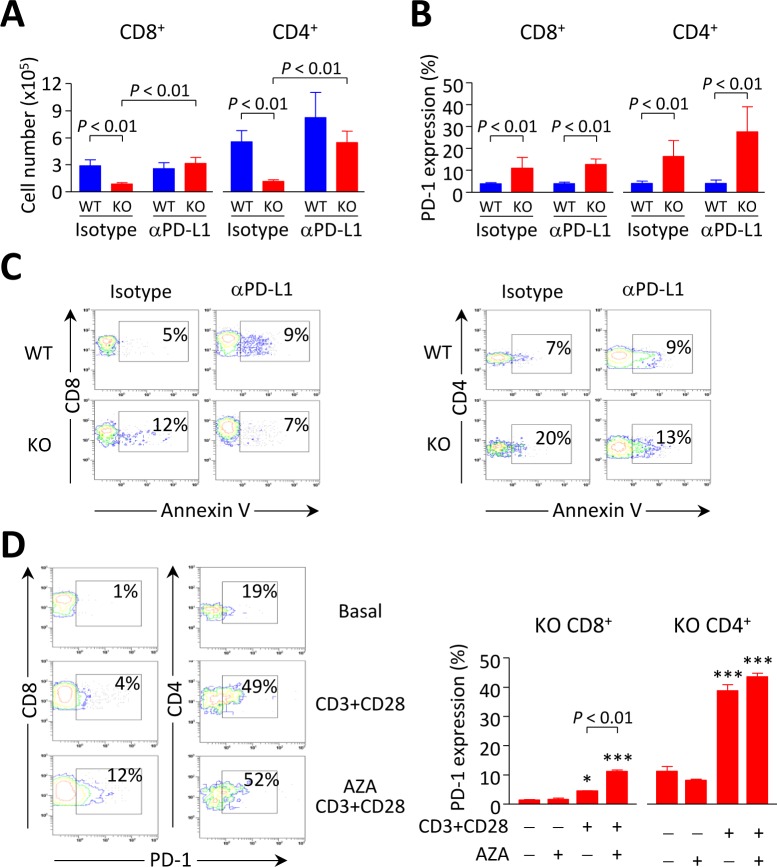
Breakdown of PD1/PD-L1 interaction contributes to accumulation of intrahepatic CD8^+^T cells in *Ae2_a,b_^−/−^* mice **A.** and **B.** Cell number (in A) and PD-1 expression (in B) on liver-infiltrating CD8^+^ and CD4^+^ T lymphocytes from *Ae2_a,b_^−/−^* and WT mice intraperitoneally injected with anti-PD-L1 mAb (120 *μ*g, five times every three days) and sacrificed at day 14. Isotype: rat IgG-isotype control. Data (in both A and B) are shown as mean ± SEM of *n =* 3 mice per genotype and group. **C.** Representative contour plots showing apoptotic rates (measured by annexin-V staining) of liver-infiltrating CD8^+^ (left) and CD4^+^ T cells (right). **D.** Representative contour plots (left) and percentages of PD-1 expression (right) on intrahepatic CD8^+^ and CD4^+^ T cells from aged *Ae2_a,b_^−/−^* mice upon stimulation with anti-CD3/CD28 Abs for three days in the presence or absence 1 *μ*M 5-aza-2′-deoxycytidine. Data (mean ± SEM) are pooled from 2 independent experiments done in triplicates. *P < 0.05, and ***P < 0.001 *versus* unstimulated condition.

We assessed whether PD-1/PD-L1 interaction might cause deletion of activated intrahepatic T cells in young *Ae2_a,b_^−/−^* mice by treating these animals with neutralizing anti-PD-L1 mAb. Intraperitoneal injections with anti-PD-L1 (but not with rat IgG-isotype control) normalized the number of intrahepatic T cells in young knockouts up to values similar to those in WT littermates (Figure 8A). Anti-PD-L1 administration also decreased the apoptotic rate without affecting PD-1 expression in intrahepatic T cells (Figure 8B, 8C). These findings point to PD-1/PD-L1 interaction as a major cause of the decrease in intrahepatic T cells observed in young *Ae2_a,b_^−/−^* mice. In aged knockouts, however, the expansion of CD8^+^ T lymphocytes in the liver may proceed without hindrance due to the aforementioned downregulation of PD-1 in these cells.

### Age-dependent silencing of PD-1 in intrahepatic CD8^+^ T cells from old knockouts is mediated by epigenetic mechanisms

We tested whether PD-1 silencing specifically observed in liver-infiltrating CD8^+^ T cells of aged knockouts could be related with *PD-1* promoter hypermethylation, as this epigenetic mechanism was recently shown to repress PD-1 expression in experimental situations [20, 21]. Thus intrahepatic T cells from aged *Ae2_a,b_^−/−^* mice were stimulated *in vitro* with anti-CD3/CD28 Abs for three days with and without the demethylating agent 5-aza-2′-deoxycytidine. We then analyzed the expression of PD-1 and found a significant increase in the proportion of PD-1^+^ CD8^+^ T cells upon stimulation in the presence of 5-aza-2′-deoxycytidine compared with the low PD-1 expression in the absence of this demethylating agent (Figure 8D). In stimulated intrahepatic CD4^+^ T cells from aged knockouts, PD-1 was expressed in a high proportion of cells, being the values similar in the presence or absence of 5-aza-2′-deoxycytidine (Figure 8D).

We also tested whether the PD-1 expression in T cells could be related to changes in the pH_i_ observed in these cells. At baseline both CD8^+^ and CD4^+^ T cells showed elevated pH_i_ in aged knockouts (Supplementary Figure S1C), and the pH_i_ increase relative to the respective WT T-cell population was higher in *Ae2_a,b_^−/−^* CD8^+^ T cells (0.26±0.03 units) than in CD4^+^ T cells (0.13±0.02 units, *P* < 0.01). We then performed additional experiments to artificially modulate the pH_i_ in purified CD8^+^ and CD4^+^ T cells from young WT mice under T-cell stimulation and found that intracellular alkalinization favors PD-1 upregulation (Supplementary Figure S3). Altogether, these results suggest that the PD-1 silencing observed in the highly activated intrahepatic CD8^+^ T cells of aged *Ae2_a,b_^−/−^* mice constitutes an aging-related and T-cell specific epigenetic change rather than an effect of intracellular alkalinization.

### The decrease in Tregs in *Ae2_a,b_^−/−^* mice is an age-related event

**Figure 9:**
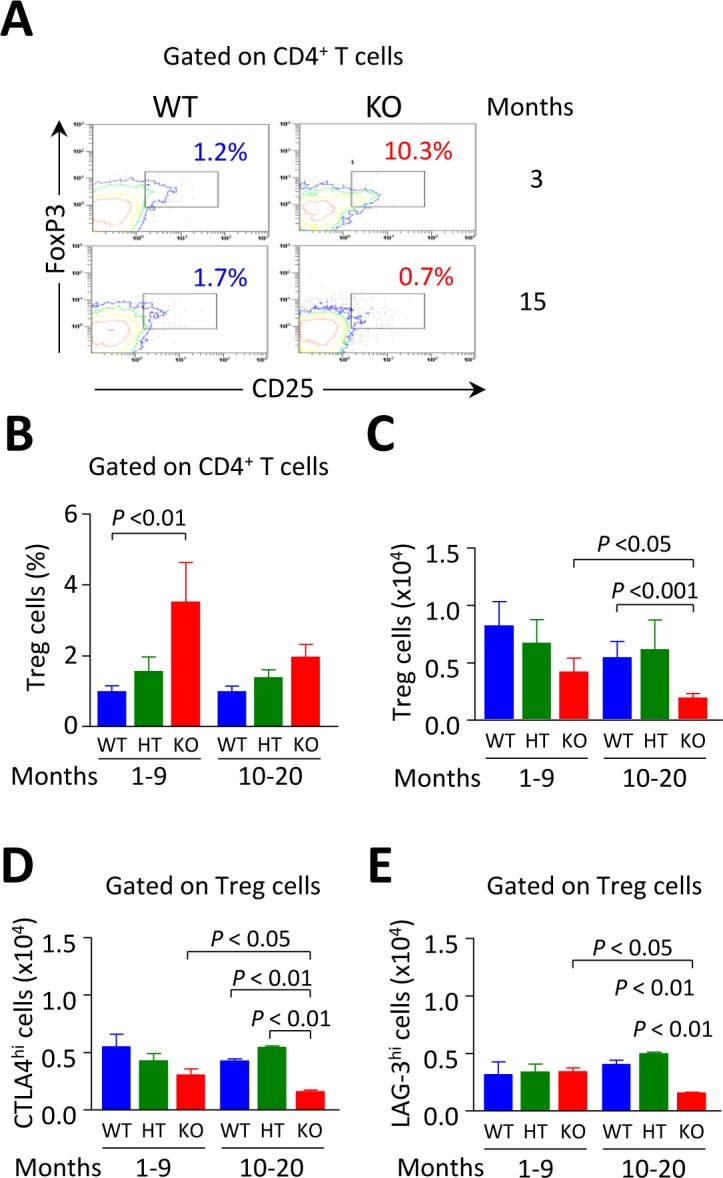
Intrahepatic Tregs are diminished in aged but not in young *Ae2_a,b_^−/−^* mice **A.** Representative contour plots showing the percentage of CD25^+^FoxP3^+^ Tregs gated on liver-infiltrating CD4^+^ T cells from young and aged *Ae2_a,b_^−/−^* and WT mice. **B.** Percentages of CD4^+^CD25^+^ Tregs gated on liver-infiltrating CD4^+^ T cells from young and aged mice of the three genotypes. **C.** Total number of intrahepatic CD4^+^CD25^+^ Tregs in young and aged mice of the three genotypes. **D.** and **E.** Total number of CTLA-4^hi^ (in D) and LAG-3^hi^ cells (in E) gated on Tregs from perfused livers of the same mice as in B and C. Shown results: mean ± SEM of *n =* 5 for young WT and HT mice, and *n =* 3 for *Ae2_a,b_^−/−^* littermates, and *n =* 4 for aged WT and HT mice, and *n =* 6 for *Ae2_a,b_^−/−^* littermates.

We have previously reported a fall of CD4^+^FoxP3^+^ Tregs in 15-month old *Ae2_a,b_^−/−^* mice [17]. Now we found that the descent of Tregs - counted as either CD4^+^CD25^+^FoxP3^+^ cells or simply CD4^+^CD25^+^ cells, since CD4^+^CD25^+^ cells are FoxP3^+^ and retain suppressor activity (Supplementary Figure S4) - is an event which evolves over time in parallel with the declined expression of PD-1 in intrahepatic CD8^+^ T cells of aged knockouts (Figure 9A-9C). This was not unexpected as the PD-1/PD-L1 system has been recognized to be essential for the induction of Tregs in the periphery [22]. Thus, we observed that the proportion of CD25^+^FoxP3^+^ Tregs was elevated within the intrahepatic CD4^+^ T-cell population in young but not in aged knockouts (Figure 9B) and that the absolute number of Tregs within the liver experienced a significant reduction when knockouts reached an advanced age (Figure 9C).

Assessment of the total number of liver-infiltrating CD4^+^CD25^+^ Tregs positive for lymphocyte-activation gene 3 (LAG-3) and cytotoxic T-lymphocyte-associated protein 4 (CTLA-4), two molecules known to play a critical role for Treg suppressor activity [23, 24], also revealed that the two suppressing subsets were only diminished in aged but not in young knockouts (Figure 9D, 9E).

**Figure 10:**
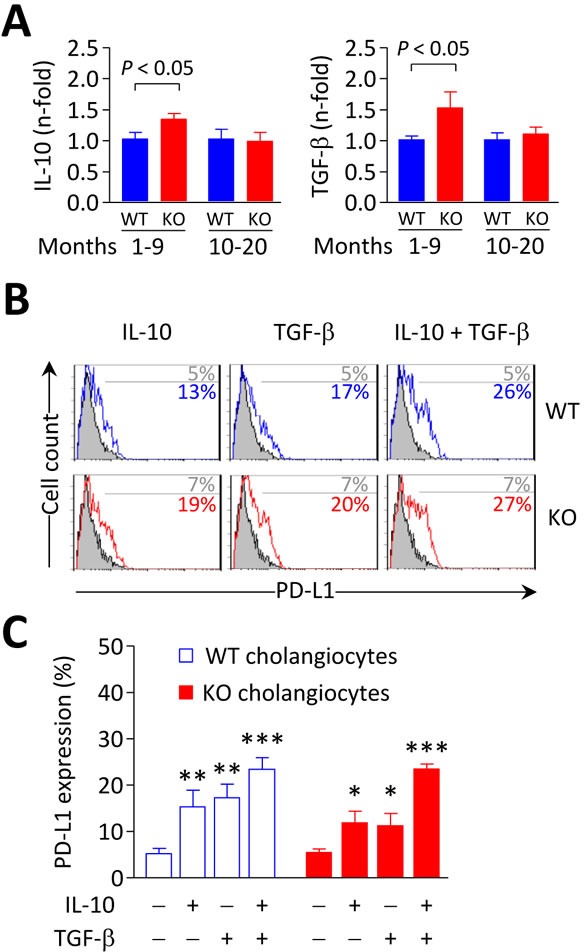
IL-10 and TGF-β induce PD-L1 expression on biliary epithelial cells **A.** IL-10 (left) and TGF-β (right) mRNA levels measured by qPCR in liver samples from *Ae2_a,b_^−/−^* and WT mice. Shown results: mean ± SEM of *n =* 5 mice per genotype and group. **B.** and **C.** Representative flow-cytometry histograms (in B) and percentages of PD-L1 expression on isolated cholangiocytes (in C) from *Ae2_a,b_^−/−^* and WT mice cultured for 2 days with 20 ng/mL IL-10 and/or 3 ng/mL TGF-β (open histograms) or with just vehicle (full histograms). Results are pooled from 4 independent experiments made by triplicates. *P < 0.05, **P < 0.01, and ***P < 0.001 *versus* basal values.

In association with these changes, the mRNA levels of the immunosuppressive cytokines IL-10 and TGF-β were upregulated in the liver of young *Ae2_a,b_^−/−^* mice but showed values similar to controls in aged knockouts (Figure 10A). Interestingly, IL-10 and TGF-β were found to upregulate PD-L1 on cultured mouse cholangiocytes (Figure 10B and 10C). Therefore, altogether our findings suggest that immunosuppressive mechanisms are induced in young *Ae2_a,b_^−/−^* mice as a homeostatic mechanism to dampen T-cell effector activation and that these control systems fail over time facilitating the development of autoimmune biliary disease at later periods of life.

## DISCUSSION

Here we show that the autoimmune phenomena that occur in *Ae2_a,b_^−/−^* mice are developed over time as a result of a late failure of immunosuppressive mechanisms. Our findings in young knockouts indicate that T cells are activated in the liver, but undergo apoptosis due to PD-1/PD-L1 interaction, a mechanism that may efficiently prevent autoimmune tissue damage early in life. Indeed, in these animals we observed an increase in the apoptosis marker annexin V on intrahepatic CD8^+^ and CD4^+^ T cells, which runs parallel with enhanced PD-1 expression on these cells and with upregulation of PD-L1 on the cell surface of biliary epithelial cells. We found that IFN-γ, a cytokine produced by effector T cells, upregulates PD-L1 expression in cultured normal and *Ae2_a,b_^−/−^* mouse cholangiocytes. It has been previously shown that IFN-γ-mediated upregulation of PD-L1 on human cholangiocytes is capable of inducing apoptosis in a co-cultured T-cell line through PD-1/PD-L1 interaction [19]. This observation and our finding of increased IFN-γ^+^ cells among intrahepatic CD8^+^ T cells from younger *Ae2_a,b_^−/−^* mice, suggest that upregulation of PD-L1 on biliary epithelial cells in the knockouts may represent a mechanism that protects cholangiocytes from autoreactive T cells via PD-1/PD-L1 interaction and apoptotic deletion of effector T cells. This mechanism provides the most likely explanation for the absence of immune-mediated liver damage at early ages and for the reduced number of intrahepatic T cells found in young knockouts. The occurrence of PD-1/PD-L1 mediated deletion of T cells within the liver of young *Ae2_a,b_^−/−^* mice is supported by our experiment of *in vivo* treatment with neutralizing anti-PD-L1 mAb which decreased the expression of annexin V on intrahepatic T-cell populations and normalized their numbers.

Recent studies indicate that PD-L1 defends the tissues against autoimmune T-cell attack, not only by inhibiting the activation and function of autoreactive T cells, but also by promoting the development of functional Tregs [22]. Thus, a *de novo* generation of Tregs was elegantly demonstrated to occur upon a synergistic effect of PD-L1 and TGF-β [25]. In this regard, the expression of PD-L1 in the intrahepatic bile ducts at early ages in *Ae2_a,b_^−/−^* mice and the upregulated liver expression of TGF-β observed in young knockouts may promptly favor the formation of Tregs from activated CD4^+^ T cells. Compared to WT littermates, young *Ae2_a,b_^−/−^* mice indeed exhibit an elevated proportion of Tregs when gated on liver-infiltrating CD4^+^ T cells. These Tregs are positive for LAG-3 and CTLA-4, which are relevant suppressor molecules for Treg function [23, 24, 26]. Moreover, young knockouts exhibit enhanced intrahepatic expression of IL-10, which together with TGF-β are major immunosuppressive cytokines secreted by Tregs [26]. Hence our data provide support to the concept that highly supressing Tregs can be induced from intrahepatic CD4^+^ T cells as a homeostatic mechanism to dampen the activation of effector T cells. Additionally, our *in-vitro* experiments indicated that both IL-10 and TGF-β are able to upregulate PD-L1 expression in isolated cholangiocytes and point to Treg cells, IL-10, TGF-β, and PD-L1 as key inter-players of a positive feedback for safeguarding the T-cell tolerance in the liver of younger *Ae2_a,b_^−/−^* mice.

The tolerogenic status in the liver of younger knockouts becomes rapidly deranged when animals reach about 10 months of age. Thus old *Ae2_a,b_^−/−^* mice show progressive accumulation of activated CD8^+^ T cells that exhibit a potent cytotoxic phenotype and decreased expression of the inhibitory molecule PD-1. Intriguingly, PD-1 is never downregulated in intrahepatic CD4^+^ T cells of aged knockouts. In line with recent findings in different experimental situations indicating that CD8^+^ T cells may epigenetically regulate their expression of PD-1 through methylation of the encoding locus [20, 21], we observed that *in vitro* stimulation of intrahepatic CD8^+^ T cells of aged knockouts in the presence of demethylating 5-aza-2′-deoxycytidine results in upregulated PD-1 expression. This was not the case, however, for the CD4^+^ T-cell population on which following stimulation, PD-1 is consistently upregulated regardless the presence of the demethylating agent. These findings support the notion that epigenetic mechanisms are involved in the robust inhibition of PD-1 expression encountered in intrahepatic CD8^+^ (but not CD4^+^) T cells of old knockouts. We recently showed that while CD4^+^ T cells possess several potential acidifying mechanisms in addition to AE2, CD8^+^ T cells only rely on AE2 for the control of pH_i_ [16]. Due to a more pronounced intracellular alkalization upon T-cell activation, *Ae2_a,b_^−/−^*CD8^+^ T cells exhibit enhanced IL-2 signals and increased reactivity with stronger effector functions [16]. Here we found that, at baseline, and compared with respective WT controls, CD8^+^ T cells of both young and aged knockouts display a more prominent elevation in the pH_i_ than CD4^+^ T cells. Moreover, experiments of artificial modulation of the pH_i_ by incubating T lymphocytes from WT mice in media buffered at different pH during their stimulation indicated that intracellular alkalinization by itself tends to increase PD-1 expression in both CD8^+^ and CD4^+^ populations. Altogether, our data suggest that the epigenetic downregulation of PD-1 observed in the liver of aged knockouts is a CD8^+^ T-cell specific event related with aging rather than with intracellular alkalinization.

**Figure 11:**
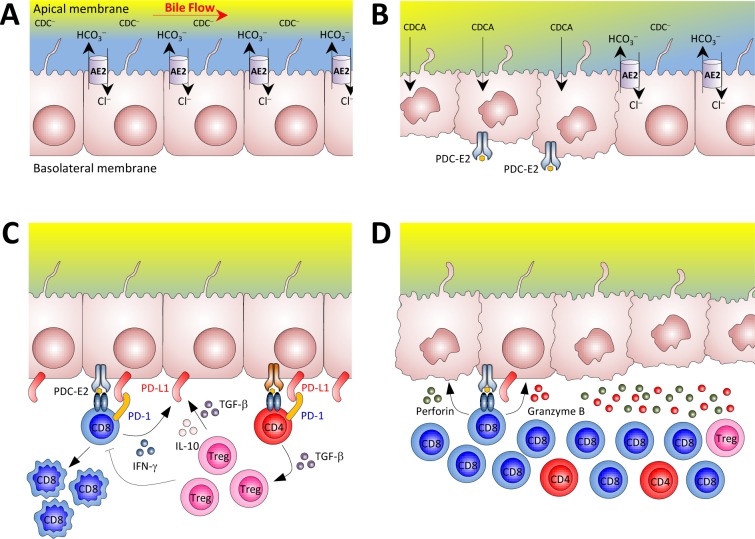
Potential mechanisms for the loss of tolerance against biliary epithelial cells in our mouse model of autoimmune cholangitis **A.** Biliary epithelial cells use AE2 for biliary HCO_3_^−^ secretion in order to develop a protective HCO_3_^−^ umbrella [42]. Biliary HCO_3_^−^ umbrella represents an alkaline environment around the luminal membrane of cholangiocytes, which prevents the protonation of hydrophobic bile salt monomers (*e.g.* chenodeoxycholic acid) -mainly conjugated with glycine in humans and with taurine in rodents- and renders them unable to permeate the cell membrane in an uncontrolled fashion, thus avoiding their toxic effects on cholangiocytes. **B.** In situations of AE2 deficiency (left side), apolar hydrophobic bile salts monomers may permeate the cell membrane of cholangiocytes and induce apoptosis. During the apoptosis process, the PDC-E2 epitope can remain intact because of the strong reduction capability of the cholangiocyte cytosol which limits adequate glutathionylation of the lipoyl moiety [43]. In a context of a “hyper-responsive” immune system, non-glutathionylated PDC-E2 peptides may promote the development of PDC-E2 specific AMA as a side-phenomenon of cholangiocyte injury. PDC-E2 epitope from the apoptotic cholangiocytes can then be recognized by circulating AMA and/or be presented by the innate immune system, further activating the adaptive immune response [44, 45]. **C.** Cholangiocytes can express different molecules linked to antigen presenting cell function [17, 46], and in PBC these cells are the direct target of the immune system. In our animal model of autoimmune cholangitis, T cells were found to be activated and deleted in the liver at early ages. Cytokines like IFN-γ, TGF-β, and IL-10 may upregulate PD-L1 expression on biliary epithelial cells which in the context of an adequate number of functional Treg cells can control self-reactive T cells. **D.** In the elderly, however, the reduced number of Tregs in the liver and PD-1 silencing in CD8^+^ T cells can make cholangiocytes more susceptible to immune attack by cytotoxic CD8^+^ T cells. *Abbreviations*: AE2, anion exchanger 2; CDC^−^; polar chenodeoxycholate; CDCA, apolar chenodeoxycholic acid; PD-1, programmed death-1; PDC-E2, E2 component of the pyruvate dehydrogenase complex.

In addition to restrained PD-1 expression other immunosuppressive mechanisms become deficient in old *Ae2_a,b_^−/−^* mice. Thus aged knockouts show reduced frequency of Tregs and deficient upregulation within the liver of inhibitory cytokines, such as IL-10 and TGF-β, despite strong activation and accumulation of CD8^+^ T cells. It is possible that the aforementioned positive feedback mechanism for intrahepatic induction of Tregs from activated CD4^+^ T cells may result disrupted in the knockouts because of progressive vanishing of biliary PD-L1 (one of the feed-back inter-players) related to subtle but continuous bile-duct destruction. *Ae2_a,b_^−/−^* cholangiocytes putatively deprived of protective biliary “HCO_3_^−^ umbrella” may be more susceptible to toxic bile acids [15]. It could be hypothesized that the absence of the “HCO_3_^−^ umbrella” not only contributes to cholangiolar damage but also to the immunogenicity of bile duct epithelial cells [27]. Thus AE2 deficiency may not only enhance the reactivity of CD8^+^ T cells because of the disturbance of their intrinsic pH_i_ regulation but, at the same time, AE2 deficiency can also promote cholangiolar injury and antigenicity (Figure 11).

The role that defective Tregs may have for PBC pathogenesis has been nicely enlightened by targeted abolition of Treg function in different animal models which develop several autoreactivity events resembling PBC [28-30]. Also, recent transfer studies based on the dnTGFβRII model emphasized that the intrinsic T-cell effector abnormality observed in that model is not sufficient to mediate autoimmune biliary disease in the setting of intact immune regulation, indicating the need of two hits [31]. Our *Ae2_a,b_^−/−^* mouse model of autoimmune cholangitis sequentially exhibit the two relevant hits, *i.e.* early activation of intrahepatic CD8^+^ T cells endowed with high cytotoxic potential, and decreased frequency of Tregs in a later stage, as reported for PBC in humans [7]. Additional agreements of our *Ae2_a,b_^−/−^* mouse model of autoimmune cholangitis with human PBC features are middle-age onset and slow progression. Moreover, a tolerogenic status may predominate early in the pathological process as suggested by the tendency to increased levels of TGF-β and PD-L1 mRNA in liver samples from patients with PBC at stages I-II as compared with late-stage samples [32].

For the progression of liver damage in PBC, there is growing evidence (both from PBC patients [4] and from most animal models resembling PBC [30, 31, 33-35]) suggesting a direct role of cytotoxic CD8^+^ T cells in biliary destruction. Our finding of downregulated PD-1 expression in CD8^+^ T cells of aged *Ae2_a,b_^−/−^* mice replicate the findings in autoreactive CD8^+^ T cells from PBC patients which upon *in vitro* CD3/CD28 stimulation displayed lower PD-1 expression and decreased apoptosis compared with the same phenotypic subset of CD8^+^ T cells from healthy controls [4]. Although large expansion of CD8^+^ T cells is not a characteristic feature of PBC patients, the human disease is accompanied by increased frequency of autoreactive CD8^+^ T cells and most genetically modified mouse models resembling PBC (including Treg-targeted models) show this phenomenon [28-30]. These Treg-targeted models may be helpful to unravel a possible mechanistic connection between the decrease in Tregs and the PD-1 downregulation in CD8^+^ T cells observed in aged *Ae2_a,b_^−/−^* mice. In any case, age-related dampening of immunosuppressive factors appears to be a key event in the development of autoimmune liver damage in our model.

In summary, we have shown that T cells are activated in the liver of *Ae2_a,b_^−/−^* mice, possibly as result of recognition of antigens expressed by dysfunctional cholangiocytes. Enhanced production of IFN-γ by effector T cells induces PD-L1 expression on cholangiocytes and deletion of reactive intrahepatic PD-1^+^ T cells through PD-1/PD-L1 interaction in young knockouts. However, PD1 is silenced in CD8^+^ T cells of aged *Ae2_a,b_^−/−^* mice and this event, together with derangement of other immunosuppressive mechanisms, allows for vigorous expansion of intrahepatic CD8^+^ T cells and autoimmune cholangitis (Figure 11) Our findings emphasize the relevance of AE2 abnormalities and age-related changes in the expression of immunoregulatory molecules for the development of autoimmune liver disease.

## MATERIALS AND METHODS

### Animals

*Ae2_a,b_^−/−^* mice (FVB/N, Balb/c, 129/Sv, and mixed background) were generated as described [36]. Each knockout was housed under pathogen-free conditions together with control littermates of the same sex. For experimentations we used both male and female mice (in agreement with our previous data in aged knockouts [17], no gender bias was hereby observed). Because of our current findings, animals were classified according to their age: younger (1-9 months old mice) and aged mice (10-20 month-old animals). All animal procedures followed the European Guidelines and were approved by the Institutional Animal Care and Use Committee of the University of Navarra.

### Isolation of mouse lymphocytes

Blood aliquots (∼200 *μ*L) were obtained from isoflurane-anesthetized mice at 1, 3, 6, 9, 12 and 15 months of age and total peripheral blood lymphocytes (PBLs) were isolated as described [16].

To isolate lymphocytes from different organs mice were also anesthetized with isoflurane and the liver was perfused through the portal vein with PBS (Gibco, Life Technologies) for about 5 minutes to wash away circulating lymphocytes. Anesthetized mice were then sacrificed and the liver, spleen and thymus were removed, weighed and processed for isolation of lymphocytes. In the case of liver-infiltrating lymphocytes, fresh liver pieces were incubated for 15 minutes at 37°C in 10 mL of RPMI 1640 + GlutaMAX (Gibco, Life Technologies) with 400 Mandl-U/mL collagenase D and 50 *μ*g/mL deoxyribonuclease I (both purchased from Roche). Digested tissues were passed through a 70-*μ*m nylon Cell Strainer (BD Falcon), and mononuclear cells were isolated in 35% Percoll gradient (GE Healthcare) at room temperature. Similar protocols were followed to isolate total lymphocytes from spleen and thymus. Briefly, freshly-extracted spleen or thymus was dissociated in complete RPMI medium supplemented with 10% fetal bovine serum (FBS), 1% penicillin-streptomycin, 1% L-glutamine and 0.1% β-mercaptoethanol, all from Gibco. After obtaining single-cell suspensions with 70-*μ*m nylon strainers, splenocytes or thymocytes were cleaned up from erythrocyte remnants by adding ACK lysing buffer (0.15 M NH_4_Cl, 1 mM KHCO_3_, 0.1 mM Na_2_EDTA, pH 7.2, all from Sigma-Aldrich), followed by centrifugation and wash with PBS, and final resuspension in MACS buffer (1x PBS with 0.5% FBS and 2 mM EDTA, pH 7.2, all from Gibco). Intrasplenic CD8^+^ and CD4^+^ T cells and CD4^+^CD25^+^ Tregs were purified in an autoMACS PRO Separator (Miltenyi Biotec) as described [16].

### Flow cytometry analysis and assessment of intracellular pH

For membrane staining and flow cytometry phenotyping analysis, 1×10^5^ lymphocytes - either isolated from different organs or from peripheral blood (PBLs) - were resuspended in MACS buffer in the presence of anti-mouse CD16/32 TruStain fcX (BioLegend, San Diego, CA) and then incubated for 15 minutes at 4°C with different combinations of fluorochrome-conjugated antibodies, including: CD3-PE-Cy7 (145-2C11), CD4-FITC (GK1.5), CD4-PE (H129.19), and CD223-PE (LAG-3, C9B7W), all purchased from BioLegend. CD8a-FITC (53-6.7), CD8a-APC (53-6.7), CD25-PE (3C7), CD44-APC (IM7), CD62L-PE-Cy7 (MEL-14), PD-1-PE (J43), and CD152-PE (CTLA-4, UC10-4F10-11) from BD Pharmingen (San Diego, CA), and CD25-APC (PC61.5) from eBioscience.

For intracellular staining of cytotoxic CD8^+^ T lymphocytes, cells were fixed and permeabilized with Cytofix/CytoPerm solution (BD Biosciences), followed by subsequent staining with antibodies to granzyme B-Alexa-Fluor 647 (GB11), interferon (IFN)-γ-PE-Cy7 (XMG1.2), both from BioLegend, perforin-PE (eBioOMAK-D, eBioscience), and CD8a-FITC (purchased as above). For intracellular staining of Tregs, cells were fixed and permeabilized with the FoxP3 Fix/Perm buffer (BioLegend), followed by staining with antibodies to FoxP3-PE (FJK-16s, eBioscience), CD4-FITC and CD25-APC (cf. above).

Apoptosis analysis was performed with the PE-Annexin V Apoptosis Detection Kit (BD Pharmingen) and an antibody cocktail with CD4-FITC and CD8a-APC (cf. above).

For flow cytometry detection of PD-L1 on bile-duct cells, normal and *Ae2_a,b_^−/−^* mouse cholangiocytes (isolated and cultured as described below), were incubated with 100 ng/mL IFN-γ, or 20 ng/mL interleukin (IL)-10 (both from ImmunoTools, Friesoythe, Germany) and/or 3 ng/mL transforming growth factor (TGF)-β (Peprotech, London, UK) for 48 hours at 37°C. Then cholangiocytes were trypsinized and stained with anti-PD-L1-APC (10F.9G2) purchased from BioLegend, in the presence of anti-mouse CD16/32 TruStain fcX.

To determine the pH_i_ of lymphocytes, total or purified CD8^+^ splenocytes were cultured in 48-well plates (1×10^5^ cells/well, each well containing 0.5 mL of complete RPMI medium) in the presence or absence of 1 *μ*g/mL anti-CD3 (BioLegend) for 1 day. Then cells were loaded with the fluorescent pH indicator 2′,7′-bis(carboxyethyl)-5,6-carboxy-fluoroscein acetoxymethyl ester (BCECF-AM; Biotium, Hayward, CA) for flow cytometry assessment of pH_i_ through the nigericin-clamp technique as described [16]. For artificial modulation of pH_i_, wildtype CD8^+^ and CD4^+^ T lymphocytes were incubated using buffered media (RPMI with 25 mM Hepes) at different pH (either 6.9, 7.4 or 7.9) as described [16]. Cells were stimulated for 1, 2 and 3 days with Dynabeads Mouse T-Activator CD3/CD28 (Life-Technologies) at 1:1 dynabead/cell ratio. The extracellular pH (pH_e_) of the respective culture media was confirmed every day and remained constant during the experiments. The changes in the pH_i_ were assessed by flow cytometry (cf. above). All the aforementioned flow cytometry analyses of lymphocytes and cholangiocytes were carried out in a Cytomics FC500 MPL flow cytometer (Beckman Coulter) with the CXP Software (Beckman Coulter).

### Treg suppression assay

A total of 1×10^5^ CD8^+^ or CD4^+^CD25^−^ splenocytes purified from WT and *Ae2_a,b_^−/−^* mice were stimulated with Dynabeads Mouse T-Activator CD3/CD28 at 1:5 dynabead/cell ratio in the presence (or absence) of 2.5×10^4^ CD4^+^CD25^+^ cells (Tregs) isolated from the spleen of the same mice. After 3 days in culture CD8^+^ and CD4^+^ T-lymphocyte populations were counted by flow cytometry.

### *In-vivo* inhibition of PD-1/PD-L1 interaction

*Ae2_a,b_^−/−^* mice and WT littermates were injected intraperitoneally with 120 *μ*g endotoxin-free PD-L1 antibody (10F.9G2, BioXCell) on day 0 and every three days up to day 12. Mice injected with rat IgG-isotype control antibody (Sigma) were used as a control group. At day 14, anesthetized mice were sacrificed and liver-infiltrating lymphocytes were isolated and analyzed by flow cytometry.

### *In-vitro* DNA demethylation assay

Lymphocytes (3×10^5^ cells) obtained from perfused livers were cultured with Dynabeads Mouse T-Activator CD3/CD28 for 3 days at 1:1 dynabead/cell ratio in the presence of 1 *μ*M 5-aza-2′-deoxycytidine (Sigma), freshly prepared before incubations. PD-1 expression on the cell surface of intrahepatic T cells was assessed by flow cytometry.

### Isolation and culture of mouse cholangiocytes

Normal and *Ae2_a,b_^−/−^* mouse cholangiocytes were prepared from intrahepatic bile-duct units isolated essentially as described [17, 37-39]. Briefly, mice were anesthetized and the portal vein was cannulated to perfuse the liver for 5 minutes with Ca^2+^- and Mg^2+^-free Hanks' buffer with 0.02% EGTA, followed by 10 minutes perfusion with Hanks' containing Ca^2+^ and Mg^2+^ and supplemented with 0.5 mg/mL of collagenase type IV (all from Sigma) and 15 mg/mL of soybean trypsin inhibitor (Invitrogen). The liver was extracted and manipulated (always under sterile conditions) in the last perfusion medium in order to separate the intrahepatic biliary tree, followed by incubation (30 minutes at 37°C, and with continuous shaking) in DMEM/F12 with Glutamax medium supplemented with 0.34 mg/mL pronase, 0.25 mg/mL collagenase type IV, 60 *μ*g/mL DNase (all from Sigma), 3% FBS and 1% penicillin/streptomycin (both from Gibco-Invitrogen). This was followed by subsequent filtrations with 100-*μ*m and 40-*μ*m nylon Cell Strainers (BD Falcon), the last filtered solution being discarded. The small tissue pieces retained in the 40 *μ*m-filter were collected and treated for 30 minutes at 37°C in the latter medium but replacing the pronase by 0.26 mg/mL hyaluronidase (Sigma). Finally, bile-duct pieces were re-filtered with the 40 *μ*m-filter and the retained material was collected and resuspended in 2-3 mL of fully supplemented Dulbecco's modified Eagle medium/F-12 medium (Gibco-Invitrogen) [40] for seeding on several wells of a 6-well Cell Culture Plate (Costar Corning Inc.) coated with 2-mm thick monolayer of rat tail collagen type I (BD). Cholangiocytes, grown in a monolayer fashion as clusters derived from the isolated bile-duct units, were passaged at 90% confluence with Dulbecco's modified Eagle medium/F-12 supplemented with 0.75 mg/mL collagenase IV and 1.5 mg/mL dispase (Invitrogen) for 10-15 minutes at 37°C followed by 6 washes with PBS. If contaminating fibroblasts were observed when the medium was exchanged every 2 days, additional treatment with just dispase was carried out followed by PBS washes. Pure cholangiocytes with positive staining for the phenotypic marker cytokeratin (CK)-7 were obtained by passages 3-4, their differentiated phenotype being maintained for over 15 passages.

### Western blotting

Mouse cholangiocytes cultured in 6-well plates were scraped in RIPA buffer (50 mM TRIS-HCl pH 7.4, 150 mM NaCl, 1% Triton X-100, 0.5% sodium deoxycholate, 0.1% SDS, 5 mM EDTA, 1 mM EGTA and 10 mM NaF -all purchased from Sigma- as well as 1 mM PMSF and complete protease inhibitor cocktail, both from Roche). Protein extracts were loaded on a precast NuPAGE Bis-Tris gel with 4-12% gradient (Invitrogen), electrophoresed and transferred to a nitrocellulose membrane. After blocking, membranes were incubated with antibodies against PD-L1 (1:1000, 10F.9G2, BioXCell) or GAPDH (1:2000, GA1R, Aviva Systems Biology). After final incubations with respective HRP-conjugated secondary antibodies, immunoreactive bands were detected by enhanced chemiluminescence (ECL, GE Healthcare) in an Odyssey Fc Western Blot Detection System (Licor Bioscience).

### Liver immunohistochemistry

Sections from formalin-fixed, paraffin-embedded liver specimens were stained for PD-L1 using 1:50 diluted anti-mouse PD-L1 rabbit IgG (Ab58810; Abcam, Cambridge, UK), anti-rabbit EnVision System-HRP (DakoCytomation, Glostrup, Dennmark), and Diaminobenzidine substrate-chromogen (DAB; DakoCytomation). For CK-7 detection in cultured mouse cholangiocytes, cells seeded on coverslips were fixed with 2% paraformaldehyde and further permeabilized with 0.1% Triton X-100. Cell preparations were stained for 1 hour at 37°C with 1:400 diluted anti-CK-7 mouse mAb IgG_1_ (RCK105, Santa Cruz Biotech) and then washed 3 times with PBS containing 1% BSA. Respective horseradish peroxidase (HRP)-conjugated secondary antibody was used at 1:400 dilution during 1 hour at 37°C. After washing as above, cells were finally treated with DAB solution (DakoCytomation) 10 minutes at room temperature. Images were acquired in an Axio Imager M1 microscope (Zeiss).

### Gene expression analysis

Retrotranscription of total RNA isolated from the liver and cultured cholangiocytes with TRI Reagent (Sigma-Aldrich) was followed by qPCR (on a StepOnePlus, Life Technologies) to determine the levels of the mRNAs for PD-L1, PD-L2, IL-10, and/or TGF-β1 (see primer pairs in Supplementary Table S1). Values were normalized for the housekeeping gene glyceraldehyde 3-phosphate dehydrogenase (*Gapdh*). Calculations were according the Livak/Schmittgen's method [41], though estimating an average amplification efficiency of 80%, i.e. 1.8^−ΔΔCT^.

### Statistical analysis

Data are expressed as mean ± SEM. Variables normally distributed according to Kolmogorov-Smirnov or Shapiro-Wilks tests, were analyzed by Student's *t-*test for comparisons between two groups, and one-way analysis of variance (ANOVA) and subsequent Bonferroni *posthoc* test for comparisons between more than two groups. Kruskal-Wallis and Mann-Whitney tests were used as nonparametric methods. Statistical analyses were performed with GraphPad Prism 5 statistical packages. A value of two-tailed *P* < 0.05 was considered statistically significant.

## SUPPLEMENTARY MATERIAL TABLE AND FIGURES


