# Supplementary material for: CD8+ T cells undergo activation and programmed death-1 repression in the liver of aged Ae2a,b−/− mice favoring autoimmune cholangitis
Source: Oncotarget. 2015 Sep 15;6(30):28588–606. doi: 10.18632/oncotarget.5665 (PMC4745679; doi:10.18632/oncotarget.5665)
Supplement: Supplementary file 1 [file oncotarget-06-28588-s001.pdf]

CD8^{+} T cells undergo activation and programmed death-1 repression in the liver of aged *Ae2*$_{a,b}^{-/-}$ mice favoring autoimmune cholangitis

Supplementary Material

Supplementary Table S1. Specific primers designed for real-time qPCR.

Mouse transcript	Oligonucleotides
PD-L1	5’- GAGTATGGCAGCAACGTCAC-3’ (sense)<br>5’- AGTTCATGCTCAGAAGTGGC-3’ (antisense)
PD-L2	5’- TGCCGATACTGAACCTGAGC-3’ (sense)<br>5’- GGAATCTCTCACTTGGACAC-3’ (antisense)
IL-10	5’- TGCTGCCTGCTCTTACTGAC -3’ (sense)<br>5’- AGTAACCCTTAAAGTCCTGC-3’ (antisense)
TGF-β1	5’- CGGCAGCTGTACATTGAC-3’ (sense)<br>5’- TCAGCTGCACTTGCAGGAGC-3’ (antisense)
Gapdh	5′-CCAAGGTCATCCATGACAAC-3′ (sense)<br>5′-TGTCATACCAGGAAATGAGC-3′ (antisense)

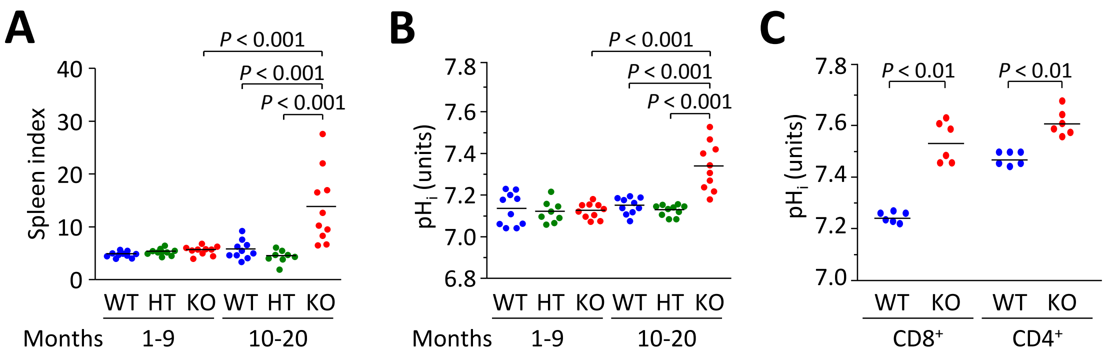

Supplementary Figure S1: *Ae2*$_{a,b}^{-/-}$ mice showed alterations in the spleen index and pH$_i$ of splenocytes over time. **A** and **B** Spleen index (in **A**) and pH$_i$ of total splenocytes (in **B**) in young and aged WT, HT, and *Ae2*$_{a,b}^{-/-}$ mice. **C** pH$_i$ of CD8$^+$ and CD4$^+$ T splenocytes from aged WT and *Ae2*$_{a,b}^{-/-}$ mice. In **A**, spleen index was calculated for each animal as the ratio of spleen weight ×1000 to body weight. Each dot represents the value for an individual mouse, and horizontal bars represent mean values.

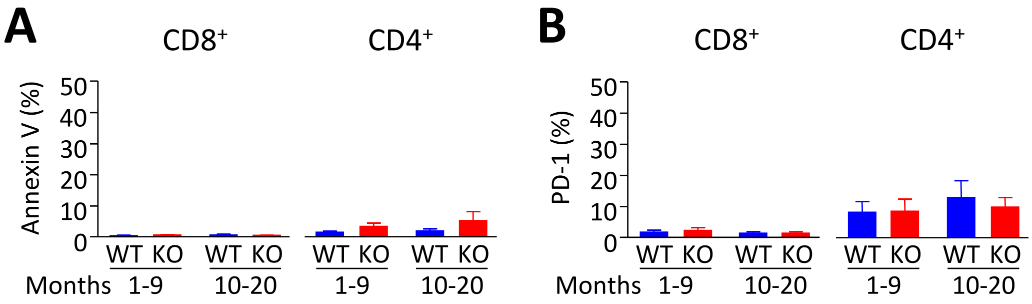

Supplementary Figure S2: Flow cytometry analysis of annexin V and PD-1 expression on PBLs. **A** and **B** Rate of apoptosis cell death measured by annexin V staining (in **A**) and PD-1 expression (in **B**) on $CD8^+$ and $CD4^+$ T cells from peripheral blood samples of $Ae2_{a,b}^{-/-}$ and WT mice. Results are shown as mean ± SEM of n = 5 mice per genotype and group.

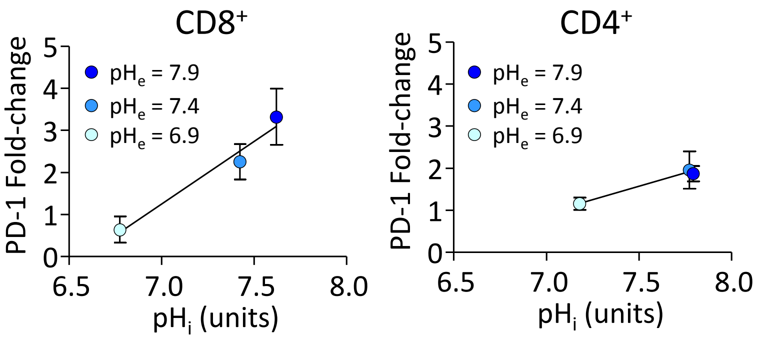

Supplementary Figure S3: Relationship between pH_i and PD-1 expression in stimulated $CD8^+$ and $CD4^+$ T cells from WT mice in media with different extracellular pH (pH_e). Cells were incubated for 3 days in complete RPMI with 25 mM Hepes buffered at pH 6.9, 7.4, and 7.9 in the presence of anti-CD3/CD28 dynabeads (at 1:1 dynabead/cell ratio). PD-1 expression and pH_i were measured by flow cytometry. Showed data are pooled from two independent experiments done in triplicates.

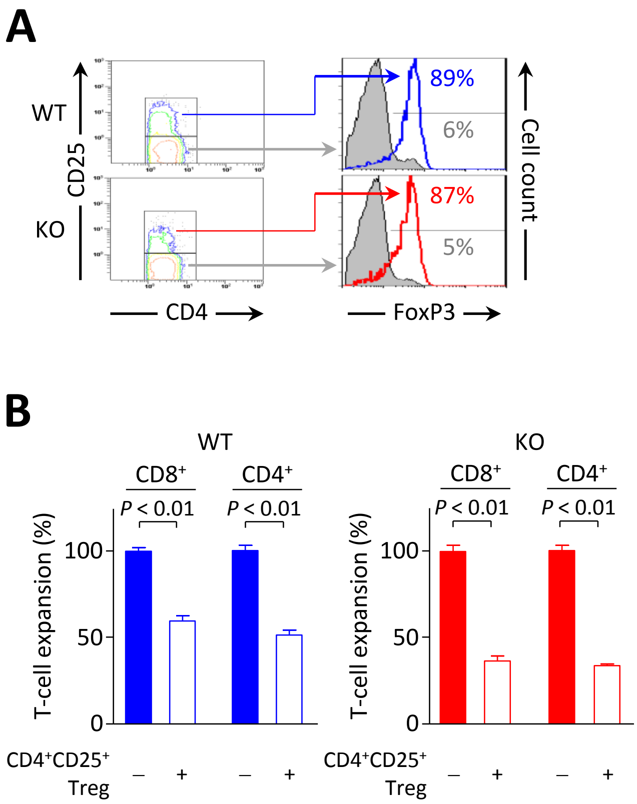

Supplementary Figure S4: CD4^{+}CD25^{+} T cells are mainly FoxP3^{+} and function as Tregs with suppressor activity on stimulated CD8^{+} and CD4^{+}(CD25^{-}) T cells. A Representative contour plot analysis (left) and flow-cytometry histograms (right) showing the 3-color gating strategy to compare the expression of FoxP3 on CD4^{+}CD25^{-} (full histograms) and CD4^{+}CD25^{+} (open histograms) cells from the spleen of WT and $Ae2_{a,b}^{-/-}$ mice. **B** CD4^{+}CD25^{+} T cells, obtained through magnetic isolation from the spleen of WT and $Ae2_{a,b}^{-/-}$ mice, behave as canonical Tregs, as they suppress the proliferation of stimulated CD8^{+} and CD4^{+}(CD25^{-}) T cells (isolated from the spleen of the respective mouse) during three days. For continuous T-cell stimulation CD3/CD28 dynabeads (at 1:5 dynabead/cell ratio) were used. Results are shown as mean ± SEM of 3 independent experiments done in triplicates.
